# Genomic Sequencing in Oncocytic Adrenal Carcinoma May Provide Insights into Disease Progression and Treatment Options

**DOI:** 10.1210/jcemcr/luaf227

**Published:** 2025-10-07

**Authors:** Hye Jeong Han, Alessandra Moore, Numbereye Numbere, Adrienne Victor, Inga Harbuz-Miller

**Affiliations:** Department of Medicine, Division of Endocrinology & Metabolism, University of Rochester, Rochester, NY 14642, USA; Department of Surgery, Division of Surgical Oncology, University of Rochester, Rochester, NY 14642, USA; Department of Pathology and Laboratory Medicine, University of Rochester, Rochester, NY 14642, USA; Department of Medicine, Division of Hematology & Oncology, University of Rochester, Rochester, NY 14642, USA; Department of Medicine, Division of Endocrinology & Metabolism, University of Rochester, Rochester, NY 14642, USA

**Keywords:** oncocytic adrenal carcinoma (OAC), adrenal cortical carcinoma (ACC), CTNNB1 mutation, Helsinki scoring system

## Abstract

Oncocytic adrenal carcinoma (OAC) is a rare histologic subtype of adrenal cortical carcinoma (ACC) characterized by >90% oncocytic cells. Unlike ACC, OACs have a reduced incidence of adrenal hormone production, larger size, and longer time to recurrence (17.5 months vs 8 months). Genetic mutations in *CTNNB1* are associated with decreased survival in ACC but their role in OAC is unknown. Here, we present a case of OAC highlighting the importance of comprehensive genomic profiling in predicting time to recurrence. A 72-year-old woman presented with rapidly progressive muscle weakness, new-onset type 2 diabetes mellitus, resistant hypertension associated with hypokalemia, and profound cognitive decline. Hormonal evaluation was consistent with ACTH-independent Cushing syndrome. A computed tomography scan of the abdomen showed a 7.1-cm heterogenous left adrenal mass. Pathology confirmed OAC after an en bloc left adrenalectomy. Next-generation sequencing revealed somatic mutations in *CTNNB1*, *CDKN2A*, and *CDKN2B*. Disease progression at 5 months after left adrenalectomy was comparable to other histopathological types of ACC, emphasizing the impact of genomic alterations in OAC.

## Introduction

Oncocytic adrenal carcinoma (OAC) is a rare subtype of adrenal cortical carcinoma (ACC). About 287 cases have been described in literature [1]. OACs are characterized by an abundant granular eosinophilic cytoplasm from accumulation of mitochondria [2]. Studies suggest that OACs have a longer time to recurrence, 17.5 months compared to 8 months in ACC [3]. The Lin-Weiss-Bisceglia (LWB) scoring system was developed to distinguish between benign and malignant oncocytic neoplasms, and the Helsinki scoring is applied to predict survival in OAC (Table 1), but they do not predict the time to recurrence [4-6]. Additionally, the Ki67 scoring methodologies and interobserver variations can limit the scoring reproducibility [7]. Genomic alterations in ACC can influence the tumor behavior (ie, *CTNNB1* mutations have a more aggressive course and alter the disease-free survival) [5]. We observed a shorter time to recurrence (5 months) in a patient with OAC harboring multiple genomic alterations in *CTNNB1* and hypothesize that genomic sequencing in OACs may serve as a potential tool to help predict disease-free survival [8].

**Table 1.** The preoperative hormonal testing result

Laboratory tested	Values	Reference range
Potassium	2.5 mmol/L (2.5 mEq/L)	3.3-5.1 mmol/L (3.3-5.1 mEq/L)
Morning cortisol	33.5 μg/dL (924.2 nmol/L)	6.0-18.4 μg/dL (165.5-507.6 nmol/L)
ACTH	<3 pg/mL (0.6 pmol/L)	7-63 pg/mL (1.5-13.8 pmol/L)
24-hour urine free cortisol	446.8 μg/day (1233.1 nmol/day)	≤45.0 μg/day (≤124.2 nmol/day)
1 mg DST	35.1 μg/dL (968.3 mol/L)	<1.8 μg/dL (<50 nmol/L).
Plasma metanephrine	<0.1 nmol/L (100 pg/mL)	<0.5 nmol/L (<500 pg/mL)
Plasma normetanephrine	0.2 nmol/L (200 pg/mL)	<0.9 nmol/L (900 pg/mL)
Plasma renin activity	0.9 ng/mL/h (0.9 mcg/L/h)	0.5-4.0 ng/mL/h (0.5-4.0 mcg/L/h)
Aldosterone	6.0 ng/dL (0.17 nmol/L)	4.0—31.0 ng/dL (0.1-0.8 nmol/L)
DHEA-S	30 μg/dL (0.8 μmol/L)	9-246 μg/dL (0.2-6.6 μmol/L)

Abbreviations: DHEA-S, dehydroepiandrosterone sulfate; DST, dexamethasone suppression test.

## Case Presentation

**Figure 1.**
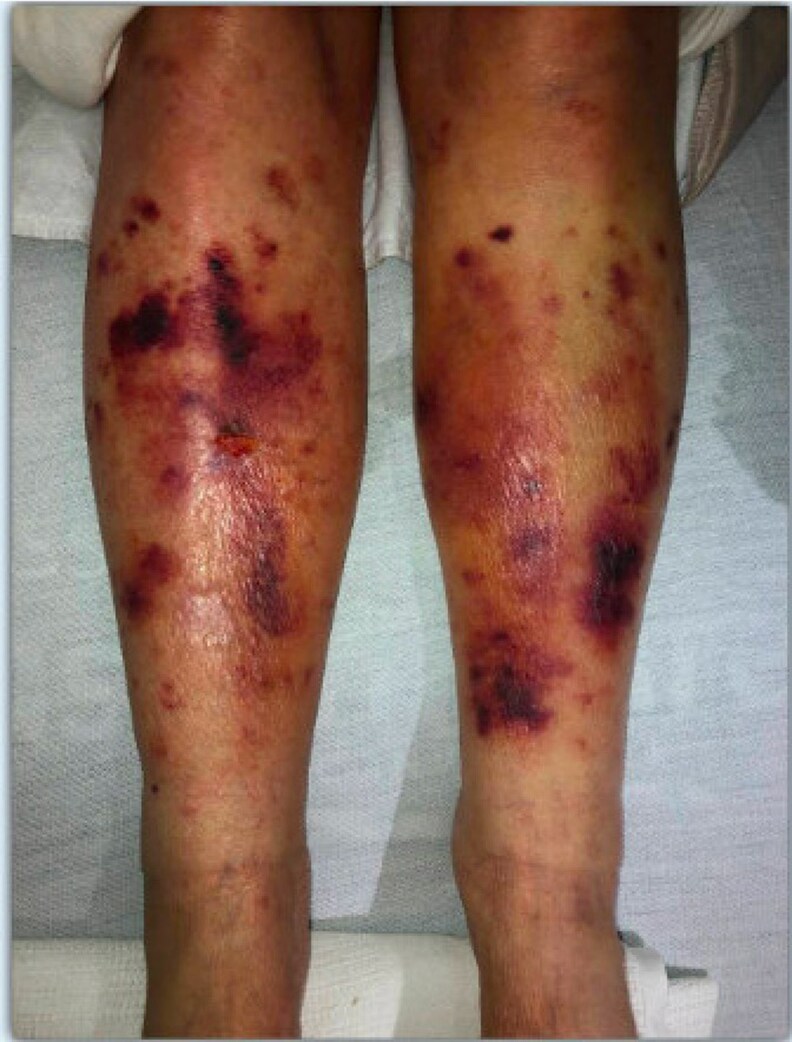
Bilateral lower extremities ecchymoses consistent with skin changes related to Cushing syndrome.

A 72-year-old woman was hospitalized with rapidly progressive muscle weakness, new-onset type 2 diabetes mellitus, resistant hypertension associated with severe hypokalemia, and cognitive decline leading to altered mental status. On physical examination, she appeared cushingoid, with a round face, supraclavicular fullness, multiple ecchymosis (Fig. 1), and proximal muscle weakness with inability to stand. She was only oriented to self; Karnofsky performance score was 30%. Her blood pressure was 184/94 mm Hg and a 4.5-kg unintentional weight gain was reported over 12 months, from baseline 63.5 kg to 68.0 kg.

## Diagnostic Assessment

The laboratory findings on admission included spontaneous hypokalemia, elevated morning cortisol with a suppressed ACTH and dehydroepiandrosterone sulfate. The 24-hour urine free cortisol was 10 times above the normal range and the 8 Am cortisol failed to suppress to 1-mg dexamethasone. Plasma metanephrines, aldosterone, and plasma renin activity were normal (Table 1).

**Figure 2.**
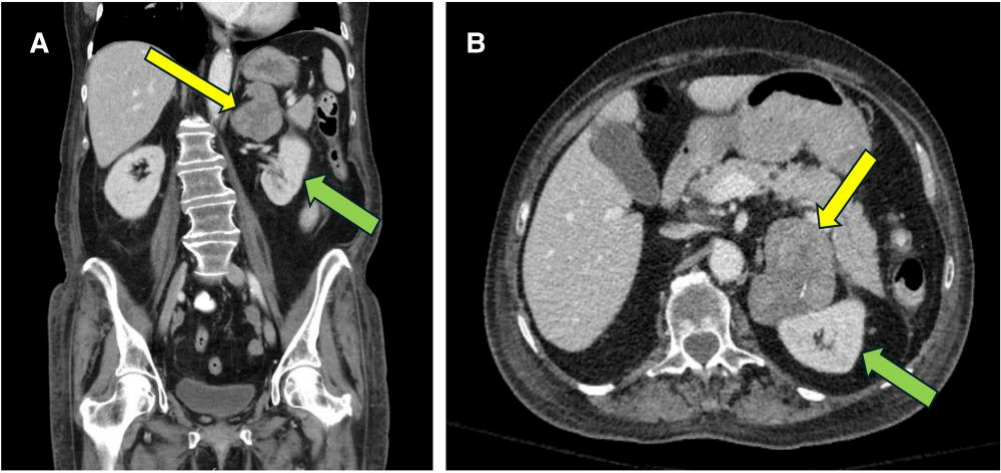
Preoperative CT of the abdomen with and without contrast in coronal view (A) and axial view (B) showing a 7.1 × 5.8 × 5.9-cm heterogenous left adrenal mass (yellow arrow). The mass was mildly heterogenous, contained a few internal calcifications, and had proximity to the left kidney (green arrow). The mass had an unenhanced attenuation of 25 Hounsfield units, 54 Hounsfield units during the venous phase, and 40 Hounsfield units postcontrast. The washout characteristics were indeterminate.

A computed tomography (CT) scan of the abdomen and pelvis with IV contrast revealed a heterogenous left adrenal mass measuring 7.1 × 5.8 × 5.9 cm (Fig. 2). The unenhanced CT attenuation of the mass was 25 Hounsfield units and washout was consistent with an indeterminate lesion. Preoperative cross-sectional images with CT of the chest, abdomen, and pelvis with IV contrast did not show evidence of metastatic lesions.

## Treatment

**Figure 3.**
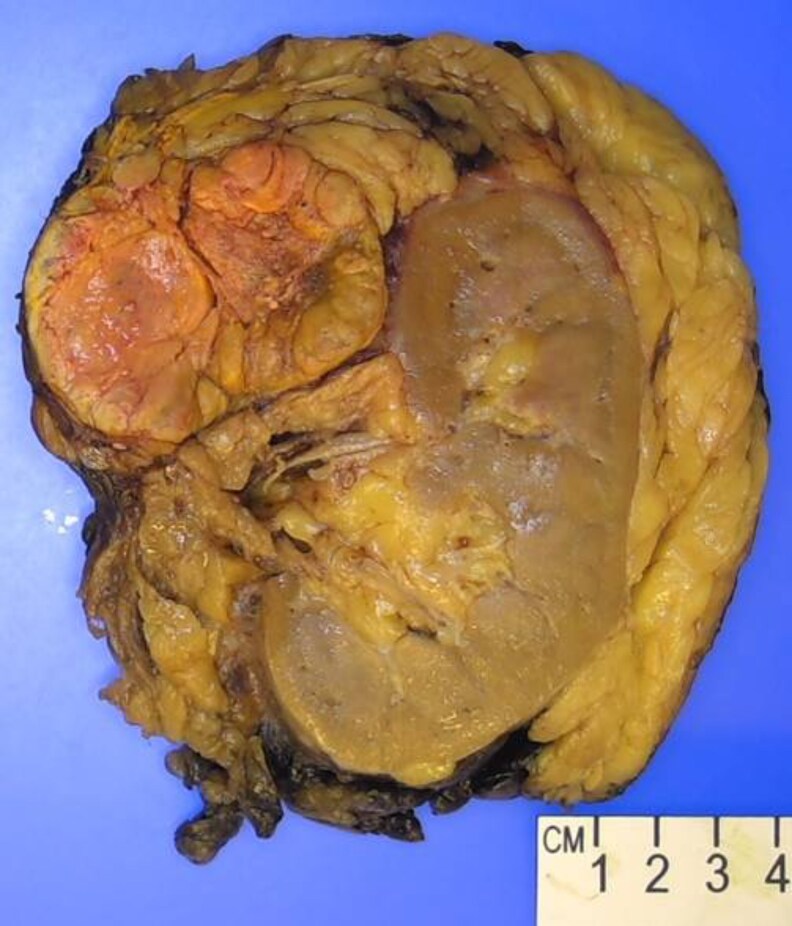
Gross photograph showing a well-circumscribed, yellow-tan adrenal cortical tumor with areas of hemorrhage and necrosis. The tumor was located superomedial to the kidney, extended into the surrounding adipose tissue, and was close to the resection margin. The adjacent kidney parenchyma appeared compressed but grossly uninvolved.

Based on radiological features, tumor size, and biochemical characteristics, a hormonally active ACC was suspected. Hypercortisolemia was controlled with metyrapone preoperatively for 2 weeks before she underwent open en bloc left adrenalectomy and left nephrectomy (Fig. 3).

**Figure 4.**
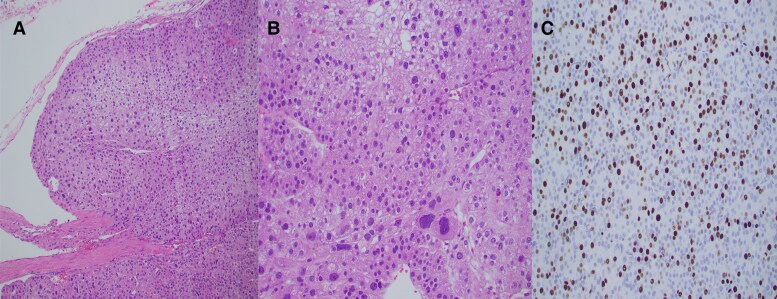
(A) (Hematoxylin and eosin [H&E], 100×): oncocytic adrenal cortical carcinoma with adrenal capsule invasion, a key feature of malignancy. The tumor was composed of large polygonal cells with abundant eosinophilic, granular cytoplasm (oncocytes), caused by an increased number of mitochondria. (B) (H&E, 200×): Marked nuclear pleomorphism was seen in this tumor—a key feature of malignancy. (C). (Ki67, 200×): The Ki-67 proliferation index of this tumor was 50%, far surpassing the 5% threshold that signifies aggressive behavior in adrenal cortical carcinomas.

Pathology revealed an oncocytic adrenal cortical carcinoma categorized as pT3N0M0. The tumor scored 7 of 7 on the Modified Weiss System with high mitotic grade of 32 per 50 high power field, clear cells comprising less than 25%, atypical mitotic figures, extensive tumor cell necrosis, and invasion through the adrenal capsule with periadrenal adipose tissue involvement. The mass extended into the adrenal vein and was less than 0.1 cm from the vein margin. Ki-67 labeling index was 50%. The SF-1 stain confirmed adrenal cortex origin (Fig. 4A-4C).

The diagnosis of OAC was based on meeting all 3 major LWB criteria: a mitotic rate of more than 5 mitoses per 50 high power field, atypical mitoses, and venous invasion. The Helsinki score was elevated at 58: 3 points for >5 mitoses per 50 high power field, 5 points for necrosis, and 50 points for the Ki-67 index (Table 2).

**Table 2.** The patient risk assessment based on modified Lin-Weiss Bisceglia criteria and the Helsinki scoring system

	Modified LWB criteria [4, 5]	Helsinki score [6]
**Criteria/score**	Major criteria: 5 mitoses per 50 high power field Any atypical mitoses Venous invasion	Mitotic count > 5 per 50 high power field (3 points) Presence of necrosis (5 points) Ki-67 proliferation index
	Minor criteria: Large size (>10 cm and/or >200 g) Necrosis Capsular invasion Sinusoidal invasion	
**Categorization**	One major criterion indicates OAC	A score of 8.5 or higher identifies metastatic potential of adrenocortical carcinoma
	One to 4 minor criteria indicate uncertain malignant potential	
	Absence of major or minor criteria indicates benign tumor	
**The scores of current patient**	All 3 major LWB criteria were positive, confirming OAC	Helsinki score 58: 3 points for >5 mitoses per 50 high power field, 5 points for presence necrosis, and 50 points for the Ki-67 index.

Abbreviations: LWB, Lin-Weiss Bisceglia; OAC, oncocytic adrenal carcinoma.

Next-generation sequencing revealed the following genomic alterations: *CDKN2A* loss of exon 1, *CDKN2B* complete homozygous deletion, and *CTNNB1* c.76C > T (p.Q26*).

## Outcome and Follow-up

Given high-risk histopathological features and concern for recurrence, the patient agreed to radiation to the tumor bed, but elected to forgo systemic therapy with etoposide, doxorubicin, cisplatin, and mitotane or mitotane alone because of concerns of adverse effects, frailty, and poor quality of life. She was treated with 25 fractions of palliative external beam radiation to the postoperative bed. The metyrapone was discontinued postoperatively and repeat 24-hour urine free cortisol normalized to 20.4 μg/day (56.3 nmol/day) (normal reference range: ≤ 45.0 μg/day; ≤ 124.2 nmol/day) at 1 month after surgery. The Karnofsky performance status improved to 50% and sensorium recovered: she was alert and oriented to self, place, and time.

**Figure 5.**
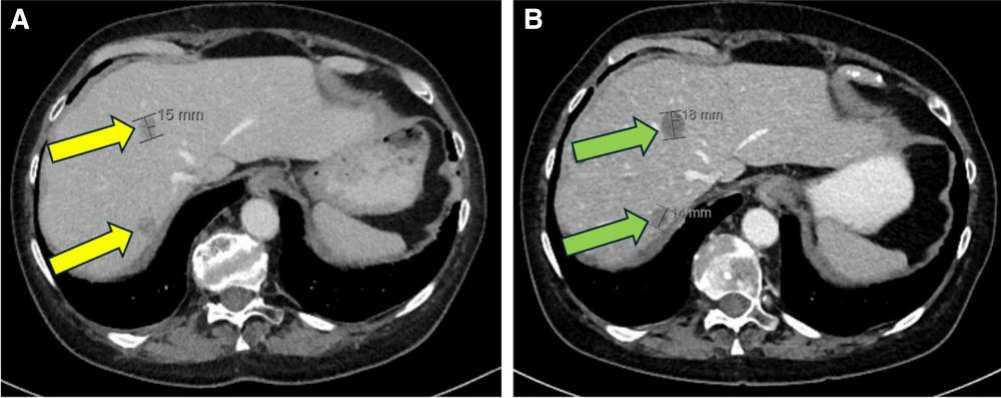
CT of the abdomen with IV contrast at 8 months (A) and at 11 months (B) after surgery revealing new hepatic lesions consistent with metastatic ACC (yellow arrows) increasing in size at 3 months interval (green arrows).

A position emission tomography/CT scan at 5 months after adrenalectomy revealed hypermetabolic bone lesions within the upper sternum, right hemi-sacrum, and left ischial tuberosity suspicious for metastatic disease that were not present when compared to prior images. The patient was treated with fractionated radiation to the bone lesions. Multiple metastatic liver lesions were noted at 8 months surveillance CT and they increased in size at 11 months postoperatively (Fig. 5A and 5B). The repeat hormonal testing revealed elevated late night salivary cortisol on 2 occasions 407 ng/dL (11.2 nmol/L) (normal reference range: <99 ng/dL; <2.7 nmol/L) and 281 ng/dL (7.8 nmol/L) (normal reference range: <99 ng/dL; <2.7 nmol/L) respectively. The patient declined conventional chemotherapy because of unacceptable adverse effects but agreed to mitotane considering hypercortisolemia. She opted to pursue pembrolizumab combined with cabozantinib because a small number of studies showed promising results of immunotherapy and tyrosine kinase inhibitor delaying disease progression [9].

## Discussion

This case highlights the limitations of the current available histopathological scoring systems and the potential use of genomic sequencing to predict time to recurrence in OAC. According to Pennanen et al, a Helsinki score >8.5 is associated with metastatic disease with 100% sensitivity and 99.4% specificity. In this study, 13 of 14 patients with ACC and a Helsinki score >8.5 had metastatic disease, although timing to disease progression was not reported. All 15 patients with ACC and a Helsinki score <8.5 had no metastatic disease during follow up ranging from 7.9 to 20.3 years [10]. Our patient had a Helsinki score of 58, correctly predicting metastatic disease with a relative short timeline to disease progression. In a study done by Cioppi et al, patients with ACC and a mutation in the Wnt/β-catenin pathway (*CTNNB1* mutation) compared to those without the mutation displayed a hazard ratio of 1.97 for disease progression. Additionally, patients with high-stage ACC and a mutation in the Wnt/β-catenin pathway showed a hazard ratio of 6.24 to progression [11]. The available literature demonstrates that OACs have greater time to recurrence of 17.5 months compared to 8 months in ACC [3]. In our case, we suspect the *CTNNB1* mutation and high Helsinki score predict a shorter time to recurrence of 5 months, underscoring the potential utility of next-generation sequencing tools and the impact of *CTNNB1* mutation on disease progression.

Additionally, genomic sequencing identified the loss of exon 1 in *CDKN2A* and loss of *CDKN2B*. These mutations have been implicated in 14.3% and 10.7% of adult stage III-IV ACC samples, respectively [12, 13]. Although the prevalence of these mutations in OAC is unknown, the mutation status may be useful when selecting adjuvant treatment option, as in vitro studies showed benefits of CDK4 and CDK6 inhibitors in ACC [14].

In summary, this case teaches us about the limitations of current histopathological scoring system and their ability to predict time to recurrence in OAC. It underscores the importance of genomic sequencing as an additional tool in guiding discussions on disease-free survival and actionable mutations for potential treatment options.

## Learning Points

- Current histopathological systems, such as the LWB criteria and Helsinki score predict malignant OAC and survival, respectively, but they do not predict time to recurrence.
- *CTNNB1* mutations have been associated with poor survival in patients with ACC, and this may extend to OAC.
- Although OAC are historically reported with more favorable prognosis than ACC, treatment and surveillance should be based on combination of histopathological features and genetics.

## Contributors

All authors made individual contributions to authorship. H.H., I.H.M., A.M., A.V.: diagnosis and management of this patient and manuscript submission. N.N.: histopathology section and preparation of histology images. A.M.: patient's surgeries. All authors reviewed and approved the final draft.

## Data Availability

Data sharing is not applicable to this article as no datasets were generated or analyzed during the current study.
